# Fine-scale GPS tracking to quantify human movement patterns and exposure to leptospires in the urban slum environment

**DOI:** 10.1371/journal.pntd.0006752

**Published:** 2018-08-31

**Authors:** Katharine A. Owers, Juliana Odetunde, Rosan Barbosa de Matos, Gielson Sacramento, Mayara Carvalho, Nivison Nery, Federico Costa, Mitermayer G. Reis, James E. Childs, José E. Hagan, Peter J. Diggle, Albert I. Ko

**Affiliations:** 1 Department of Epidemiology of Microbial Diseases, Yale School of Public Health, New Haven, Connecticut, United States of America; 2 University of Kentucky College of Medicine, Lexington, Kentucky, United States of America; 3 Fulbright-Fogarty US Scholar Program, Fogarty International Center and the National Institute of Mental Health, Salvador, Brazil; 4 Vanderbilt-Emory-Cornell-Duke Global Health Consortium Fogarty Global Health Fellowship, Fogarty International Center and the National Institute of Mental Health, Nashville, Tennessee, United States of America; 5 Centro de Pesquisas Gonçalo Moniz, Fundação Oswaldo Cruz, Ministério da Saúde, Salvador, Brazil; 6 Instituto de Saude Coletiva, Federal University of Bahia, Salvador, Brazil; 7 CHICAS, Lancaster Medical School, Lancaster University, Lancaster, United Kingdom; University of Maryland, College Park, UNITED STATES

## Abstract

**Background:**

Human movement is likely an important risk factor for environmentally-transmitted pathogens. While epidemiologic studies have traditionally focused on household risk factors, individual movement data could provide critical additional information about risk of exposure to such pathogens. We conducted global positioning system (GPS) tracking of urban slum residents to quantify their fine-scale movement patterns and evaluate their exposures to environmental sources of leptospirosis transmission.

**Methodology/Principal findings:**

We recruited participants from an ongoing cohort study in an urban slum in Brazil and tracked them for 24 hours at 30-second intervals. Among 172 subjects asked to participate in this cross-sectional study, 130 agreed to participate and 109 had good quality data and were included in analyses. The majority of recorded locations were near participant residences (87.7% within 50 meters of the house), regardless of age or gender. Similarly, exposure to environmental sources of leptospirosis transmission did not vary by age or gender. However, males, who have higher infection rates, visited a significantly larger area during the 24-hour period than did females (34,549m^2^ versus 22,733m^2^, p = 0.005). Four male participants had serologic evidence of *Leptospira* infection during the study period. These individuals had significantly larger activity spaces than uninfected males (61,310m^2^ vs 31,575m^2^, p = 0.006) and elevated exposure to rodent activity (p = 0.046) and trash deposits (p = 0.031).

**Conclusions/Significance:**

GPS tracking was an effective tool for quantifying individual mobility in the complex urban slum environment and identifying risk exposures associated with that movement. This study suggests that in addition to source reduction, barrier interventions that reduce contact with transmission sources as slum residents move within their communities may be a useful prevention strategy for leptospirosis.

## Author summary

Environmental features of urban slums including inadequate sanitation, substandard housing, and population crowding predispose residents to numerous infections. Despite this shared environment, not all slum residents, even within households, have equal risk of infection with specific pathogens and we do not know why. Individual movement data will help us better understand how slum residents interact with their environment. We conducted GPS tracking of 109 urban slum residents in Brazil to quantify their movement patterns and how these influence exposure to leptospirosis, an environmentally transmitted infection common in urban slums. Slum inhabitants, regardless of age and gender, spent most of their time close to home and had similar exposures to environmental features associated with leptospirosis infection. However, males visited a larger area on a daily basis, which may explain their higher leptospirosis risk. Based on screening of the slum population conducted at six-month intervals, four individuals (all male) became infected with *Leptospira* during our study. These individuals visited a significantly larger area than other males and had higher exposure to rodents and trash deposits than did other participants. GPS tracking allowed us to identify movement and movement-induced exposure as risk factors for leptospirosis infection and could provide similarly important information for other environmentally-transmitted pathogens.

## Introduction

Household characteristics are frequently used as a measure of the risk of pathogen exposure. However, these household risk factors are static proxies for exposure, which is a dynamic function of movement through a contaminated environment. Many neglected tropical diseases—including schistosomiasis, helminthiasis, and leptospirosis, among others—result from contact with environmental pathogens. Individual movement data can provide a more complete picture of exposure to such pathogens than can household-based proxies, and so add value to epidemiological studies [1]. The availability of ever cheaper, smaller, and more accurate location-aware devices has made it feasible to quantify movement [2]. Mobile phone data are increasingly used to study large-scale population movements [3, 4] and GPS data to study fine-scale individual activities [5–9]. Tracking technology has been applied to diseases such as malaria [4, 10], dengue fever [2, 11, 12], schistosomiasis [13, 14], and non-communicable disease [9, 15], and has yielded valuable insights into exposure and transmission. But despite these examples of effective use of movement data, knowledge of human movement patterns and their influence on exposure remains limited.

The study of environmentally transmitted disease will benefit from an improved understanding of human movement. For some diseases, pathogen sources are generally known and movement can help understand interaction with them. For others, the specific source causing human infection is unknown. Risk factor associations between infection and household characteristics can indicate that exposure occurs in the peridomestic environment. However, an important question is what mechanism these associations capture: do they indicate that the pathogen is present in the household environment or that the individual comes into contact with the pathogen at a source near the home? Specific information on how human-pathogen contacts occur is important for planning public health interventions, and movement may provide this information.

Leptospirosis is an environmentally transmitted disease [16] for which movement may play a critical role in understanding exposure risk. This spirochetal zoonosis is transmitted to humans when cut or abraded skin comes into contact with water or soil contaminated with the bacteria, which are released into the environment via the urine of an infected mammalian host [17]. While many cases are asymptomatic or mild, leptospirosis causes approximately one million hospitalized cases and nearly 60,000 deaths per year worldwide [18]. Severe cases can manifest as Weil’s disease with jaundice and renal failure or a pulmonary hemorrhagic syndrome [19], which have case fatality rates of >10% or >50% respectively [20]. Leptospirosis occurs worldwide, but its burden is highest in subsistence farming and urban slum populations in the developing world [18, 20].

Individual movement data may be particularly informative for studies of leptospirosis in the urban slum environment. Urban slums are home to a large and growing population, expected to total two billion by 2030 [21]. Leptospirosis has emerged as a major public health problem in slums worldwide because their poor sanitation and housing infrastructure promote rat-borne transmission of the pathogen [22–26]. Detailed longitudinal studies of urban slum leptospirosis in Salvador, Brazil and other locations have revealed that it manifests as seasonal, rainfall-associated epidemics [27–29]. Infection rates for males are nearly twice those for females in an urban slum in Salvador (48.3 vs 25.9 infections per 1000 follow-up events [30], see also [27, 31–33]), consistent with findings from other urban locations [34–36]. Risk factors for infection are concentrated in the peridomestic environment, where leptospirosis disproportionately affects individuals living in rat-infested households in close proximity to transmission sources such as sewers, open trash deposits, mud, and floodwaters [24, 32, 37, 38]. However, the risk of pathogen transmission varies widely over small distances [30] and there is substantial unexplained variation in risk, even within households, where only some residents are infected. Urban slums are complex, compact environments that can vary widely at small spatial scales and individual movement through this heterogeneous environment may play a key role in generating the observed risk heterogeneity. There may also be differences in movement between groups, such as males and females, with different infection rates. Movement could also help answer the question of whether leptospires are present in the peridomestic environment or whether slum residents contact the pathogen at sources near the home.

We used GPS tracking to quantify the movement patterns of urban slum residents and their resulting exposure to environmental sources of leptospires. Because urban slums are highly heterogeneous environments, movement studies must be conducted at fine spatial scales. We densely sampled participant movement, recording their location every 30 seconds for 24 hours, to obtain high spatial resolution data. We recruited participants from four demographic groups: males aged 15–34 years, females aged 15–34 years, males aged ≥35 years, and females aged ≥35 years. This recruitment scheme allowed us to describe age- and gender-specific patterns and evaluate the hypothesis that males’ increased risk of leptospirosis is due to their movement patterns and resulting exposure to environmental features associated with leptospirosis infection.

## Methods

### Study site and population

Pau da Lima is an urban slum in Salvador, the third largest city in Brazil. This slum, at the periphery of the city, occupies the floor and sides of several connected valleys. Sewers—most of which are open—flow through the valley floors, and drainage tributaries run down the sides of the valleys. Housing ranges from formal concrete block structures to dwellings constructed of found materials. Informal employment is frequent, but regular employment is available at a nearby commercial area and throughout the city, to which the slum is connected via bus. A 2012 census of the site identified 12,651 residents who were mostly squatters (88%), did not complete primary school (66%) and subsisted on a median per capita daily income of US$2.60. This site is a high-transmission setting for leptospirosis: outpatient surveillance identified a mean annual incidence for clinical disease of 143 cases per 100,000 population since 2010, and hospital-based surveillance initiated in 2001 identified 19.8 hospitalized cases per 100,000 population per year.

Participants in this GPS study are drawn from a cohort of Pau da Lima residents being followed prospectively to determine the seroincidence of leptospirosis. This cohort study, initiated in 2013, enrolls residents who sleep at least three nights a week in Pau da Lima, are at least 5 years old, and consent to participate. Researchers visit participants every six months to take blood for serological analysis and conduct interviews about demographic, socioeconomic, and behavioral features. Household exposures are determined through these interviews and geographic information systems (GIS) surveys. Samples are evaluated using the microscopic agglutination test, the gold standard serologic diagnostic test for leptospirosis [17]. Infections are defined by a seroconversion (from a titer of <1:50 to ≥1:50) or a four-fold rise in titer between sequential samples. The samples bracketing the GPS study were taken in March and September 2014.

This study, conducted from June-September 2014, focused on a high-risk sub-cohort living at low elevation in a single valley. Residents at low elevation in this site have low socioeconomic status and live near open sewers and flood-prone areas, all features associated with leptospirosis infection. GPS study participants were drawn from cohort participants at least 15 years old due to the low incidence of leptospirosis in children. We recruited participants from four groups: young males (aged 15–34 years), young females (15–34 years), older males (aged ≥35 years), and older females (aged ≥35 years). The study was approved by Institutional Review Boards at Yale University and in Brazil. All participants provided written informed consent. Consent was also obtained from parents or guardians of participants who were minors.

### Optimization of GPS protocol

We considered three commercially available GPS models (the Mobile Action Technology iGot-U 120 and 600, and the SleuthGear iTrail) and chose the Mobile Action Technology iGot-U 120 for its balance of cost and features. This model or its predecessor the iGot-U 100 was used in previous studies [7, 8, 14]. The mean battery life of our units at a 30-second interval was 46.7 hours, insufficient for 48-hour recording, leading us to focus our sampling effort on 24-hour periods. We conducted accuracy tests using three randomly selected units. These were turned on, given five minutes to establish satellite connection, then placed together on a flat surface and left to collect data every 30 seconds for 10 minutes. We repeated this procedure in locations around the study neighborhood and city and under varying meteorological conditions to produce an accuracy measure reflective of the range of conditions experienced by participants. To the GPS points taken under each testing condition, we fitted a bivariate Normal distribution with zero mean, independent components and a pooled standard deviation. We used the mean pooled standard deviation across testing conditions, 5.67 meters, hereafter referred to as SD_ACC_, in our analyses.

Movement is sensitive information, and we took steps to ensure participants were comfortable with our GPS study. We benefitted from the extensive work conducted by Paz-Soldan *et al*. [6] in Iquitos, Peru, where they implemented a GPS study to study the role of movement in dengue virus infection. We created an information sheet modeled after theirs that explained why we were studying movement, how this would help us understand leptospirosis, and what participation entailed. It also answered common questions including what the GPS could and could not record, that there are no known health risks associated with the technology, and what would happen if the unit was lost, stolen, or broken (participants had no responsibility for these incidents). Sheets contained both written and visual versions of the information due to the high illiteracy rate in the study site. We used this information sheet during recruitment and gave participants a copy so they could explain the study to others. We conducted several pilot studies of the GPS enrollment and wearing procedure, updating the protocol and information sheet to address participant questions.

### Sampling design and enrollment

We recruited participants through door-to-door sampling (the method used to enroll participants in the source cohort study). To avoid biasing our sample by employment status, we recruited throughout the day and on all days of the week. We recruited one individual per household at a time to avoid accidental switching of GPS units. Each recruitment week, we took 20 GPS units into the field with the goal of recruiting five people from each of the four demographic groups. We evaluated recruitment numbers weekly, and if any groups were under-represented, we preferentially recruited members of that group when encountered.

When we met an eligible individual, we gave a verbal introduction to the project complemented by the written and illustrated information sheet. We allowed them to examine the GPS unit and ask questions about the study and technology, then asked if they were willing to participate. If not, we recorded the reason(s) for refusal. If they consented, we completed a consent form and entry interview to collect demographic data, then gave them the GPS unit and instructions. Sixty-one participants were also enrolled in an activity diary study. The diary, conducted retrospectively when we returned to pick up the GPS unit, asked about the participant’s activities, behaviors, and exposures each hour of their 24-hour tracking period.

### Data collection

GPS units were delivered to participants after being programmed to turn on one hour before the desired start time of data collection (to ensure that the unit was on and connected to satellites by that time) and off at the end of collection. Units were set to record the GPS location every 30 seconds. Settings were not modifiable by participants. We instructed participants to wear the units around the neck on the attached ribbon, a method found to minimize clothing-related interference and maximize acceptability to study participants [2]. Participants were told when to start and stop wearing the GPS unit. We arranged a time to come back to collect it and asked that participants leave it with someone in their house if they could not be there at the set time.

At the end of the assigned 24-hour period, we returned to the house, retrieved the GPS unit, and conducted an exit interview to gather data on compliance with study protocols and answer questions that arose during participation. If the participant was enrolled in the activity diary study, we also completed the diary at this time. We downloaded the GPS data using the unit’s software (@TripPC, http://www.a-trip.com) to a secure server (REDCap) and erased the unit’s memory. Data were formatted and analyzed in R version 3.2 [39] and visualized in QGIS version 2 [40].

### Data quality control and cleanup

We used exit interviews to categorize participants as fully, partially, or non-adherent to the study protocol. We then evaluated the length of data collection (affected when units either suffered early battery exhaustion or started recording data after the programmed start time) and density of data collection (a function of the actual inter-point interval, which was programmed to be 30 seconds). We tested whether the amount of missing data varied by age, gender, household elevation and per-capita income, GPS unit, and where the unit was worn (the neck, as instructed, or other locations as reported by participants during their exit interview).

We trimmed data to the desired start and end times, and excluded self-reported time when the participant did not wear the unit. Each GPS point included the time, date, latitude, longitude, and distance from previous point. We calculated the time interval and velocity (distance divided by interval) between points. We also implemented a one meter per second velocity filter, excluding points with velocities above this threshold. This filter removes points with obviously incorrect recorded locations while discarding minimal data [13]. It serves an additional purpose in our study by excluding points when participants were moving too fast to be traveling by foot. Because leptospirosis is transmitted by contact with contaminated soil or water, we did not consider time in motorized transport to be at-risk.

### Analysis of movement patterns

We first measured the concentration of participant activity near the house and within the neighborhood. We retrieved the GPS coordinates of participant households from our cohort study data and calculated the distance of each GPS point from the wearer’s house. We defined the neighborhood as the valley in which the study was conducted, and used a geographic information system shapefile of that valley’s boundary to determine whether each GPS point was in or outside the neighborhood.

We then calculated the area visited in 24 hours (activity space) using the Daily Path Area (DPA) method from Zenk *et al*. [9]. The activity space is quantified by buffering an individual’s GPS points then calculating the total area contained within the buffer. This buffering step can account for positional inaccuracy as well as movement between data points. This is important for diseases like leptospirosis where exposure can occur anywhere an individual contacts the environment, not just at their GPS points. The distance between the recorded GPS point and the wearer’s actual location is a function of variation along both the north-south and east-west axes, and we modeled this using a bivariate Normal distribution. This induces a Rayleigh distribution for the distance between recorded and actual locations, which requires a radius of 2.45 times the standard deviation to capture with 95% probability the actual location of the GPS point. We calculated the activity space using two different buffer radii to evaluate the sensitivity of the measure. We first used a radius of 2.45*SD_ACC_ that accounts largely for positional uncertainty of the GPS point. We then tested a 2.45 SD_ACC_ + 20 meter buffer to incorporate both positional uncertainty and space potentially visited while the participant was walking in the interval between points.

### Exposure due to movement

We next measured exposure to leptospirosis transmission sources resulting from participant movement. Previous studies in our site and others have identified a number of household environmental features associated with leptospirosis infection [30–33, 37, 38]. In this study we evaluated exposure to open sewers and trash deposits, low elevation (a proxy measure for flood risk), land cover (vegetated, exposed soil, or impervious surface), and rodent activity. Open sewers and public trash deposits were delineated through environmental surveys. Elevation was extracted from a one-meter elevation contour of the site. We used remote sensing to classify each two-meter pixel on a high-resolution satellite image of our site as vegetation, exposed soil, or impervious surface [41]. Tracking plates, which have been shown in this site to be a sensitive measure for quantifying rodent activity [42], were used to generate a fine-scale map of predicted rodent activity [41]. Several of these exposure measures require intensive field surveys and so were only available within the study site, not the entire city. Exposure analyses were thus restricted to movement within the site.

To quantify exposure, we generated a grid of points with 2.5 meter spacing that covered the study neighborhood. We then assigned each grid-point a value for each exposure. These values were defined as: the reciprocal of the squared distance to sewer or trash, the closest 1-meter elevation contour, binary values for presence of each land use class at the grid point, and predicted rodent activity at the grid point. We then overlaid each GPS point on the grid and used a Gaussian kernel smoother with formula–*exp[(distance between grid point and GPS point)*^*2*^ / (2* SD_*ACC*_^*2*^*)]* to calculate that GPS point’s weighted mean for each exposure based on nearby grid-points. We calculated the mean exposure to each environmental feature across a participant’s full set of GPS points. Because of this spatially smoothed estimation, exposure values are best interpreted as a relative measure, not a directly interpretable quantity.

## Results

### Study sample

The study area contained 402 eligible individuals, of whom we contacted 172 during the study period and enrolled 130 (75.6%, Table 1). Contact rates were slightly higher for females but enrollment rates are similar across age and gender. Participants are generally similar to the total slum population except that they are significantly more likely to live within 10 meters of an open sewer (p < 0.001). This difference is expected due to our focus on high-risk households along the valley bottom.

**Table 1:** Characteristics of the source urban slum population (“eligible”) and study participants (“enrolled”), by age and gender group.

	Males, 15–34 years old		Females, 15–34 years old		Males, ≥ 35 years old		Females, ≥ 35 years old	
	Eligible	Enrolled	Eligible	Enrolled	Eligible	Enrolled	Eligible	Enrolled
**Count (Acceptance Rate**^1^**, %)**	n = 82	n = 25 (78.1%)	n = 131	n = 48 (75.0%)	n = 83	n = 26 (83.9%)	n = 106	n = 31 (68.9%)
**Age, median (IQR)**	24 (18.25–30)	23 (17–28)	25 (19–30)	25 (19–29)	46 (41–58)	50 (41–62)	46 (39–52)	43 (38.5–55)
**Per capita daily income $US, median (IQR)**	2.29 (1.50–3.00)	1.81 (1.09–2.47)	1.43 (0.31–2.45)	1.25 (0.26–2.39)	2.57 (1.41–4.35)	2.69 (2.19–4.63)	2.00 (1.06–3.50)	1.61 (0.85–2.56)
	**Literate (%)**	96.3	96.0	94.5	93.4	87.3	88.5	71.7	64.5
**Self-reported Black race (%)**	56.1	60.0	46.6	54.2	31.1	27	53.8	67.7
**House flooded in the last 6 months (%)**	8.3	13.0	8.1	12.8	14.3	20.8	7.1	14.3
**House within 10m of open sewer (%)**^2^	62.7	92.0	75.2	95.8	70.4	83.3	68.9	89.3
**Trash deposits <10m from home (%)**	19.4	21.7	22.5	25.5	17.1	16.7	18.8	17.9
**Employed (%)**	68.8	68.0	34.4	35.4	80.6	76.9	55.5	54.8

^1^ The acceptance rate is calculated from the number contacted per group (left to right): 32, 64, 31, 45

^2^Significant difference between the eligible and enrolled populations (p < 0.0001)

### GPS data quality measures

The most common reason for refusal to participate was being too busy. Of 130 participants, 100 (76.9%) wore the GPS unit for the entire assigned time, 11 (8.5%) complied partially with instructions and could report times when they were non-adherent, and 19 (14.6%) were considered entirely non-adherent. Forgetting to wear the unit was the main reason for both full and partial non-adherence. For two fully adherent participants, no data were recorded by the GPS unit. Analyses were thus conducted on 109 participants (98 fully and 11 partially adherent participants whose data was censored during their self-reported non-adherent time).

The length of data collection was generally high, but the density was lower than expected. Early battery exhaustion or late recording start were both relatively rare, and 87 participants (79.8%) had GPS points spanning more than 90% of the assigned 24 hours. The median interval between GPS points was 35 seconds, with 90.8% under 1 minute and 99.6% under 5 minutes. The GPS records of eleven individuals (10.1%) included at least 75% of the expected 2880 points, and 65 (59.6%) included at least 50%. Females had more missing data than males (p = 0.041). The amount of missing data did not vary by age of the wearer (p = 0.742), GPS unit (p = 0.198), where individuals wore the GPS unit (neck versus other location, p = 0.602), or per-capita income (p = 0.840) or elevation (p = 0.191) of the wearer’s household.

### Movement and exposure among study participants

**Fig 1:**
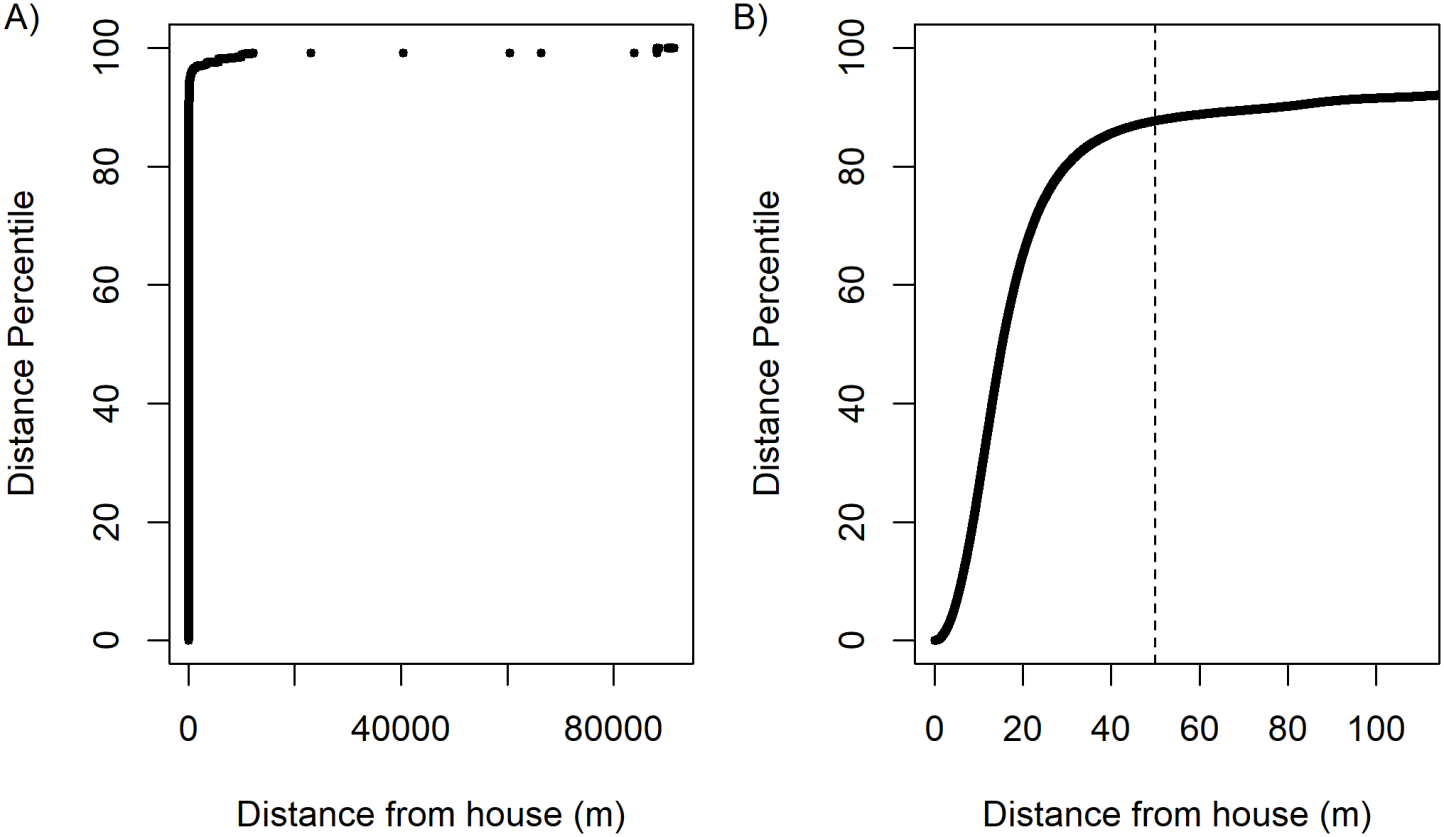
Distances of GPS points from the wearer’s house. We calculated the distance between each GPS point and the wearer’s home and converted these distances into percentiles. While the maximum distance is approximately 88,000 meters (A), zooming in on the 100 meters closest to the house (B) indicates that the majority of points occur near the home. The dashed line marks 50 meters, within which 87.7% of all GPS points fall.

Certain movement characteristics are similar across age and gender, but others differ by demographic group. Regardless of age or gender, participants spent most of their time near their residence. A median of 94.9% of points occurred within the study site and 87.7% within 50 meters of the home (Fig 1). In contrast, activity space, the entire area encompassed by an individual’s movement, does vary significantly by age and gender. Males visit a much larger space on a daily basis than females (mean 34,549m^2^ versus 22,733m^2^, p = 0.005), as do older participants compared to younger (mean 31,739m^2^ versus 23,111m^2^, p = 0.033). Results are reported for the 2.45 SD_ACC_ radius, but are qualitatively similar for the 2.45 SD_ACC_ + 20 meter radius. While most time is spent in the peridomestic environment, males and older adults move more within that space. In addition, behavioral data recorded in activity diaries for 61 (56.0%) of the GPS participants showed that males reported spending nearly three more hours per day outdoors than females (mean 4.95 versus 2.28 hours, p = 0.002). This is consistent with males visiting a larger area in a 24-hour period.

We also estimated movement-induced exposure to environmental features associated with leptospirosis infection. Interestingly, we did not identify any features for which exposure varied by age or gender (Table 2), despite variation in activity space. Though males move through a larger area and spend more time outside, their exposures are similar to those of other groups.

**Table 2:** Movement-induced exposure to leptospirosis transmission sources.

	OPEN SEWERS	TRASH DEPOSITS	ELEVATION	VEGETATED	EXPOSED SOIL	IMPERVIOUS SURFACE	RODENT ACTIVITY
**MALES 15–34 YEARS OLD**	32.36 (11.813)	0.00106 (0.00038)	32.349 (0.626)	0.245 (0.05)	0.249 (0.023)	0.507 (0.049)	-4.184 (0.062)
**FEMALES 15–34 YEARS OLD**	37.099 (8.250)	0.00465 (0.00164)	31.398 (0.619)	0.178 (0.028)	0.218 (0.016)	0.604 (0.034)	-4.246 (0.052)
**MALES AGED ≥35 YEARS**	21.247 (5.681)	0.00233 (0.00163)	33.033 (0.950)	0.125 (0.025)	0.202 (0.026)	0.673 (0.043)	-4.285 (0.055)
**FEMALES AGED ≥35 YEARS**	20.015 (6.741)	0.00127 (0.00034)	33.041 (0.716)	0.223 (0.032)	0.217 (0.022)	0.559 (0.046)	-4.354 (0.059)
**P-VALUE**	0.347	0.161	0.260	0.111	0.596	0.095	0.247

The mean (standard error) exposure to each feature during 24 hours. Exposure values were assigned to a grid of points with 2.5 meter spacing that covered the entire study area. Each GPS point’s exposure value was calculated using a Gaussian kernel centered on the GPS point and summarizing the exposure values of nearby gridpoints. Exposure measures are best interpreted as a relative quantity.

### Movement and exposure among infected participants

Four participants had serologic evidence of leptospirosis infection during the 6-month interval containing the GPS study. This infection prevalence of 3.7% (4/109) is similar to the 2.9% (47/1600) in the full cohort during the study period (p = 0.563). All infected individuals were male, with two in each age group (15–34 and ≥35 years). Infected participants are generally similar to all male participants except on ethnicity—infected individuals all self-identified as Black, compared to 47.5% of all male participants (p = 0.035).

**Fig 2:**
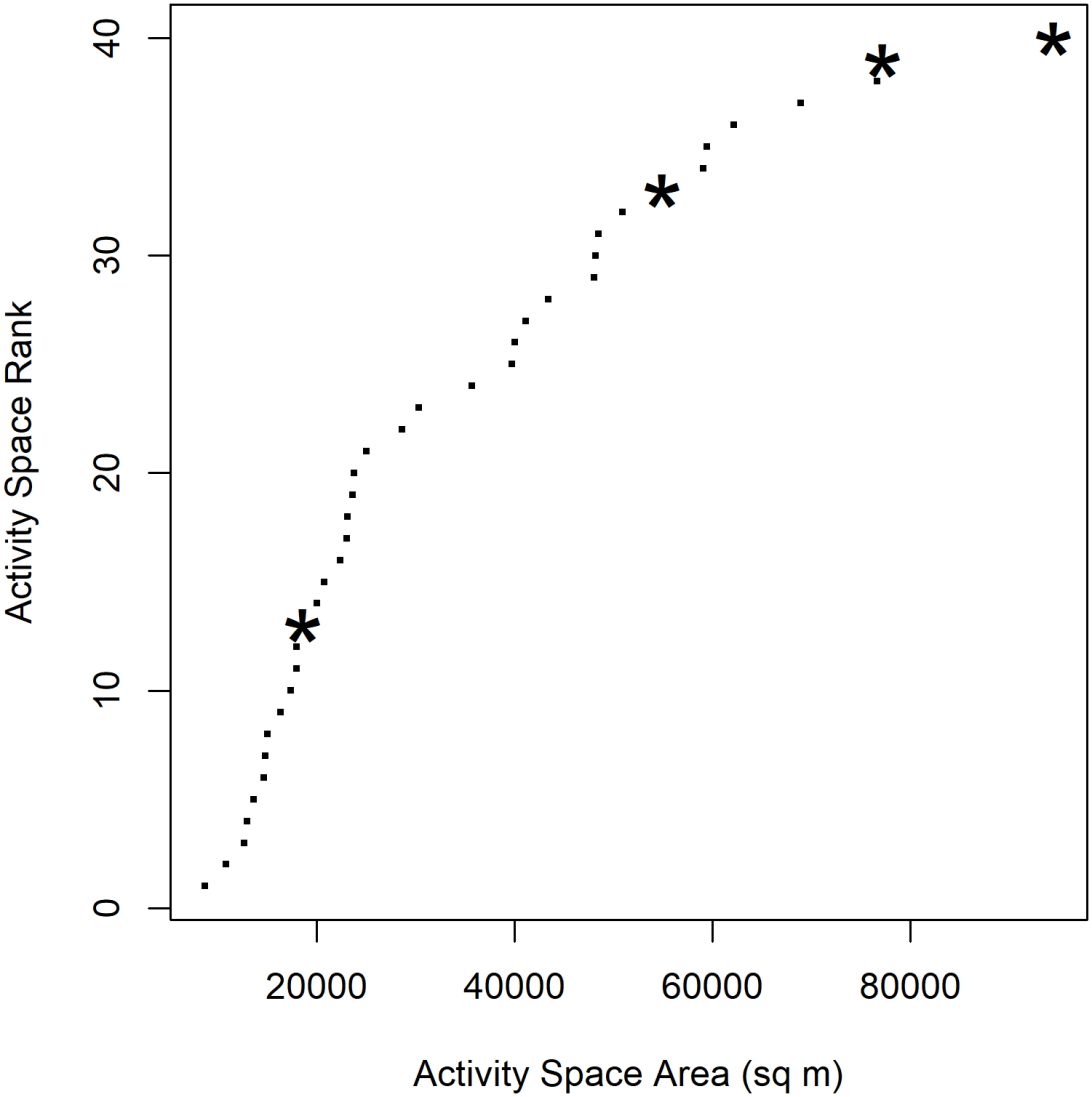
Activity space of infected males compared to all males. The activity spaces of all males in our study (n = 40) were ranked by area. The four infected individuals are marked with asterisks.

Both the movement and exposure of infected individuals showed unique features. Because males have larger activity spaces than females, we compared the activity spaces of infected males (n = 4) to those of uninfected males (n = 36). Infected individuals had three of the ten largest activity spaces (Fig 2) and a significantly larger mean activity space than uninfected males (61,310m^2^ vs 31,575m^2^, p = 0.006). While we did not identify associations between exposure and specific risk groups at the population level, we did in infected individuals. Exposure to both trash and rodent activity were significantly higher in infected individuals than in other study participants (see Table 3).

**Table 3:** Movement-induced exposure to leptospirosis transmission sources, by infection status.

	OPEN SEWERS	TRASH DEPOSITS	ELEVATION	VEGETATED	EXPOSED SOIL	IMPERVIOUS SURFACE	RODENT ACTIVITY
**UNINFECTED**	28.878 (4.348)	0.00232 (0.00064)	32.404 (0.374)	0.190 (0.017)	0.219 (0.011)	0.591 (0.022)	-4.281 (0.029)
**INFECTED**	19.201 (10.014)	0.01084 (0.00802)	30.503 (1.851)	0.243 (0.140)	0.248 (0.075)	0.509 (0.127)	-4.023 (0.187)
**P-VALUE**	0.543	**0.031**	0.816	0.271	0.305	0.744	**0.046**

The mean (standard error) exposure to each feature during 24 hours. Exposure values were assigned to a grid of points with 2.5 meter spacing that covered the entire study area. Each GPS point’s exposure value was calculated using a Gaussian kernel centered on the GPS point and summarizing the exposure values of nearby gridpoints. Exposure measures are best interpreted as a relative quantity.

## Discussion

Human mobility data can more accurately capture interactions with the environment than can household-based risk proxies. The technological barriers to quantifying movement have lowered and movement data are increasingly being used in infectious disease research. Movement has been studied among low socioeconomic status residents of cities in the developed world [e.g. 9, 15] and in isolated cities and areas in the developing world [e.g. 2, 8, 13]. However, we previously knew little about the movement patterns of slum residents in large cities in the developing world and how these patterns may affect exposure to the many diseases for which they are at risk, including leptospirosis. We implemented GPS tracking in an urban slum in Brazil and identified both strengths and limitations of the method. Quantifying movement allowed us to identify the area visited in a day as a novel risk factor for leptospirosis. We also note that specific movement-induced environmental exposures are higher in infected individuals, whereas such associations are absent at the population level. GPS tracking of urban slum residents proved an effective way to study their movement patterns and resulting exposure to a contaminated environment. This technology is suitable for use in future studies on leptospirosis and other environmentally transmitted pathogens.

### GPS optimization and limitations

Steps taken to optimize our GPS protocol were successful, resulting in few refusals or problems with non-adherence. Our selected GPS model, the MobileAction iGot-U 120, was reasonably priced yet programmable, durable, and password protected. High participant acceptance rates indicated that privacy concerns do not preclude GPS studies in this setting. This high acceptance rate may be due in part to our group’s familiarity to the community, but residents found the technology interesting and were eager to wear the units, an attitude which may transfer to other locations. Additionally, other groups have had success implementing GPS studies in diverse settings [8, 9, 13].

GPS tracking has limitations that must be considered when determining the method’s suitability for specific research questions and settings. First, GPS locations are not exact. The standard deviation of our units’ recorded locations was 5.67 meters in each coordinate direction, and even more expensive higher-accuracy units have errors on the order of meters. In complex environments with fine-scale environmental heterogeneity, exposure can vary at smaller distances than this. Second, GPS points are not taken exactly as scheduled, and data can contain gaps of several hours (often associated with participants going indoors). Third, the GPS unit will record data regardless of whether it is being worn as instructed. Finally, recorded movement patterns may be atypical, either due to unusual circumstances or a conscious effort to modify one’s behavior due to observation. In the absence of directly observing GPS participants, researchers must rely on self-reported adherence to study instructions of wearing the GPS during a day of normal activity.

We were able to avoid or adjust for these limitations in the current study. By buffering GPS points in the activity space analyses we took into account both participant movement between points and point location error. Similarly, in our exposure analyses, we used a spatial kernel to take a weighted average of exposure values near the recorded GPS point to account for positional uncertainty. Exposure to leptospires likely occurs outdoors, so GPS points lost while participants were indoors should not affect relevant exposure measures. Finally, by conducting careful exit interviews, we were able to elicit information about non-adherence to study protocols.

### Movement patterns and leptospiral exposure

Population-level analysis revealed that participants spent most of their time near their home and that exposure to environmental features associated with leptospirosis infection was similar across age and gender groups. People living in households near trash deposits, open sewers, vegetation and exposed soil, areas of high rodent activity, and flood-prone areas have an elevated risk of leptospirosis infection [32, 37, 38]. However, when we examined movement-induced contact with these environmental features, we did not identify a specific exposure associated with males, the group at highest risk of infection. It seems that within the peridomestic environment where residents spend most of their time, exposure to these features is ubiquitous and does not differentiate between risk groups. It is possible that our focus on high-risk individuals living in the valley bottom obscured differences that would be observed in the total slum population, but our study population was representative of the total population on all metrics except household proximity to an open sewer. Though we did not find associations between specific environmental exposures and leptospirosis risk, these results highlight the importance of the peridomestic environment, an area with dense exposure to transmission sources where people spend most of their time, as a critical location for public health action. Interventions in this setting will improve conditions experienced during a high proportion of slum residents’ time.

While males spend as much time near the home as females, over 24 hours males visit a significantly larger area within the peridomestic environment. Thus, activity space identified the population group at high risk (males). Males have higher employment rates than females, but within each gender activity space does not significantly differ by employment status (p = 0.182 for males and 0.184 for females). That males have larger activity spaces seems therefore to be a difference between genders which is unrelated to their employment status. This daily travel through a larger area may place males at higher risk of infection by exposing them to more potential transmission sources, some of which by chance impart a sufficient inoculum dose.

Analysis of the four infected individuals revealed unique movement and exposure characteristics. Infected males had significantly larger activity spaces than the average male and some of the largest activity spaces among all participants, lending further support to the hypothesis that movement is a risk factor for infection. Additionally, infected individuals had significantly elevated exposure to rodent activity and to trash deposits, which provide rodent habitat and food. These associations hint that movement generates exposure to bacteria excreted in the environment by rodents, the reservoir of urban leptospirosis.

Our results indicate that household-based risk factors capture movement of individuals to bacteria at sources near the household, instead of general peridomestic contamination. If household proxies captured risk due to pathogens in the household environment, infection rates should be equal across age and gender groups according to our time-near-home and exposure analyses. But instead, movement behaviors captured by the activity space are associated with infection. Public health measures that construct barriers to movement-induced contact with environmental bacterial sources may therefore be an important additional intervention against leptospirosis.

### Limitations & conclusions

This study tracked a small number of high-risk urban slum residents over a single 24-hour period per person. Because we tracked a subset of participants on each sampling day, we could not robustly examine temporal interactions between individuals. We therefore focused on general movement and exposure patterns instead of shared interactions or specific locations visited. The study population lives closer to sewers than the total urban slum population but is otherwise representative, indicating that our findings about where slum residents spend time and how much they move are generalizable across this site. Despite our relatively small sample size of 109, we identified four infections and detected associations in the population as a whole as well as among the four infected individuals. Our finding that males have larger activity spaces may be influenced by the fact that females have more missing data. However, males also report spending nearly three more hours per day outdoors on average than do females. This is consistent with males having both a higher number of GPS points and a larger activity space, so we feel that our study captured a true difference in behavior by gender. Finally, the battery life of our units limited us to 24 hours of tracking at a time. While we tracked each individual once to maximize population coverage, future studies could track individuals over several days to estimate inter-day variation in movement and exposure. Slum resident movement patterns likely vary according to specific features of the urban context, but the finding that individuals spend much of their time near home may be generalizable to other populations with low levels of formal employment. The association between movement and infection risk may apply to other diseases for which the causative agent is present in the environment.

In conclusion, GPS tracking is an effective tool for understanding movement and exposure in urban slum populations. By tracking urban slum residents for a 24-hour period, we identified features of their movement patterns and resulting exposures that will inform further studies of leptospirosis as well as preventive measures. While it is important to consider methodological limitations of GPS tracking during study design, individual movement could provide critical additional information for studies of the broad range of environmentally-transmitted pathogens, both in urban slums and other settings.

## Supporting information

S1 Checklist STROBE checklist. (DOC)

S1 File Anonymized dataset of participant characteristics and GPS compliance, technical function, and tracking results. Data dictionary included.
(XLSX)
