# Supplementary material for: Fine-scale GPS tracking to quantify human movement patterns and exposure to leptospires in the urban slum environment
Source: PLoS Negl Trop Dis. 2018 Aug 31;12(8):e0006752. doi: 10.1371/journal.pntd.0006752 (PMC6143277; doi:10.1371/journal.pntd.0006752)
Supplement: S1 Checklist — (DOC) [file pntd.0006752.s001.doc]

STROBE Statement—Checklist of items that should be included in reports of ***cross-sectional studies***

|  | Item No | Recommendation |
| --- | --- | --- |
| **Title and abstract** | 1 | (*a*) Indicate the study’s design with a commonly used term in the title or the abstract Abstract, Methodology/Principal Findings |
|  |  | (*b*) Provide in the abstract an informative and balanced summary of what was done and what was found  Abstract, Methodology/Principal Findings |
| Introduction | | |
| Background/rationale | 2 | Explain the scientific background and rationale for the investigation being reported  Introduction, paragraphs 1-4 |
| Objectives | 3 | State specific objectives, including any prespecified hypotheses  Introduction, paragraph 5 |
| Methods | | |
| Study design | 4 | Present key elements of study design early in the paper  Methods, paragraphs 2-3 |
| Setting | 5 | Describe the setting, locations, and relevant dates, including periods of recruitment, exposure, follow-up, and data collection  Methods, paragraphs 1-3 |
| Participants | 6 | (*a*) Give the eligibility criteria, and the sources and methods of selection of participants  Methods, paragraphs 2-3 |
| Variables | 7 | Clearly define all outcomes, exposures, predictors, potential confounders, and effect modifiers. Give diagnostic criteria, if applicable  Methods, paragraphs 2, 8-15 |
| Data sources/ measurement | 8* | For each variable of interest, give sources of data and details of methods of assessment (measurement). Describe comparability of assessment methods if there is more than one group  Methods, paragraphs 12-15 |
| Bias | 9 | Describe any efforts to address potential sources of bias  Methods, paragraph 6 |
| Study size | 10 | Explain how the study size was arrived at  Results, paragraph 1 |
| Quantitative variables | 11 | Explain how quantitative variables were handled in the analyses. If applicable, describe which groupings were chosen and why  Methods, paragraphs 13, 15 & Introduction, paragraph 5 |
| Statistical methods | 12 | (*a*) Describe all statistical methods, including those used to control for confounding  Methods, paragraphs 10-15 |
|  |  | (*b*) Describe any methods used to examine subgroups and interactions  NA |
|  |  | (*c*) Explain how missing data were addressed  Methods, paragraphs 10-11 |
|  |  | (*d*) If applicable, describe analytical methods taking account of sampling strategy  NA |
|  |  | (*e*) Describe any sensitivity analyses  Methods, paragraph 13 |
| Results | | |
| Participants | 13* | (a) Report numbers of individuals at each stage of study—eg numbers potentially eligible, examined for eligibility, confirmed eligible, included in the study, completing follow-up, and analysed  Results, paragraphs 1-2 & Table 1 |
|  |  | (b) Give reasons for non-participation at each stage  Results paragraph 2 |
|  |  | (c) Consider use of a flow diagram |
| Descriptive data | 14* | (a) Give characteristics of study participants (eg demographic, clinical, social) and information on exposures and potential confounders  Table 1 |
|  |  | (b) Indicate number of participants with missing data for each variable of interest  NA |
| Outcome data | 15* | Report numbers of outcome events or summary measures  Results, paragraphs 4-7 |
| Main results | 16 | (*a*) Give unadjusted estimates and, if applicable, confounder-adjusted estimates and their precision (eg, 95% confidence interval). Make clear which confounders were adjusted for and why they were included  NA |
|  |  | (*b*) Report category boundaries when continuous variables were categorized  NA |
|  |  | (*c*) If relevant, consider translating estimates of relative risk into absolute risk for a meaningful time period  NA |
| Other analyses | 17 | Report other analyses done—eg analyses of subgroups and interactions, and sensitivity analyses  Results, paragraphs 6-7 |
| Discussion | | |
| Key results | 18 | Summarise key results with reference to study objectives  Discussion, paragraphs 1, 5-7 |
| Limitations | 19 | Discuss limitations of the study, taking into account sources of potential bias or imprecision. Discuss both direction and magnitude of any potential bias  Discussion, paragraphs 3-4, 9 |
| Interpretation | 20 | Give a cautious overall interpretation of results considering objectives, limitations, multiplicity of analyses, results from similar studies, and other relevant evidence  Discussion, paragraphs 1, 10 |
| Generalisability | 21 | Discuss the generalisability (external validity) of the study results  Discussion, paragraph 9 |
| Other information | | |
| Funding | 22 | Give the source of funding and the role of the funders for the present study and, if applicable, for the original study on which the present article is based  Additional information, Funding |

*Give information separately for exposed and unexposed groups.

**Note:** An Explanation and Elaboration article discusses each checklist item and gives methodological background and published examples of transparent reporting. The STROBE checklist is best used in conjunction with this article (freely available on the Web sites of PLoS Medicine at http://www.plosmedicine.org/, Annals of Internal Medicine at http://www.annals.org/, and Epidemiology at http://www.epidem.com/). Information on the STROBE Initiative is available at www.strobe-statement.org.
